# A Latent Class Analysis of Multimorbidity and the Relationship to Socio-Demographic Factors and Health-Related Quality of Life. A National Population-Based Study of 162,283 Danish Adults

**DOI:** 10.1371/journal.pone.0169426

**Published:** 2017-01-05

**Authors:** Finn Breinholt Larsen, Marie Hauge Pedersen, Karina Friis, Charlotte Glümer, Mathias Lasgaard

**Affiliations:** 1 DEFACTUM - Public Health & Health Services Research, Central Denmark Region, Aarhus, Denmark; 2 Research Centre for Prevention and Health, Capital Region of Denmark, Glostrup University Hospital, Glostrup, Denmark; 3 Department of Psychology, Southern University of Denmark, Odense, Denmark; Fraunhofer Research Institution of Marine Biotechnology, GERMANY

## Abstract

**Objectives:**

To identify patterns of multimorbidity in the general population and examine how these patterns are related to socio-demographic factors and health-related quality of life.

**Study design and setting:**

We used latent class analysis to identify subgroups with statistically distinct and clinically meaningful disease patterns in a nationally representative sample of Danish adults (N = 162,283) aged 16+ years. The analysis was based on 15 chronic diseases.

**Results:**

Seven classes with different disease patterns were identified: a class with no or only a single chronic condition (59% of the population) labeled “1) Relatively Healthy” and six classes with a very high prevalence of multimorbidity labeled; “2) Hypertension” (14%); “3) Musculoskeletal Disorders” (10%); “4) Headache-Mental Disorders” (7%); “5) Asthma-Allergy” (6%); “6) Complex Cardiometabolic Disorders” (3%); and “7) Complex Respiratory Disorders” (2%). Female gender was associated with an increased likelihood of belonging to any of the six multimorbidity classes except for class 2 (Hypertension). Low educational attainment predicted membership of all of the multimorbidity classes except for class 5 (Asthma-Allergy). Marked differences in health-related quality of life between the seven latent classes were found. Poor health-related quality of life was highly associated with membership of class 6 (Complex Cardiometabolic Disorders) and class 7 (Complex Respiratory Disorders). Despite different disease patterns, these two classes had nearly identical profiles in relation to health-related quality of life.

**Conclusion:**

The results clearly support that diseases tend to compound and interact, which suggests that a differentiated public health and treatment approach towards multimorbidity is needed.

## Introduction

Better living conditions, scientific advances, and technological improvement in healthcare allow a significant proportion of the population to survive diseases that were previously fatal; and as a result, a growing proportion of the population is reported to have multimorbidity [1] which is here defined as the presence of two or more chronic diseases in the same individual [2, 3]. This development has been reinforced by intensified chronic disease screening and diagnosing. Since the risk of chronic diseases increases significantly during the life course, multimorbidity can be expected to become even more prevalent in the future due to the aging of the population.

The high prevalence of multimorbidity is one of the main challenges facing governments and healthcare systems around the world. The main reasons for this are that in most countries the healthcare system is configured primarily for individual diseases rather than for multimorbidity, and that guidelines for care usually take a single-morbidity approach [4]. In patients with multimorbidity, a single-disease focus as opposed to an integrated approach may heighten the risk of iatrogenic harm, causing undesirable sequelae and increasing the risk of complex drug interactions and side effects due to polypharmacy [5]. Furthermore, multimorbidity is associated with a lower quality of life, functional decline, increased disability, fragmentation of care, a greater treatment burden, and higher mortality [6–10].

Detailed knowledge of the epidemiology of multimorbidity lies at the root of any attempt at tailoring the healthcare system to the need for treating a growing number of people with multiple, chronic conditions. However, multimorbidity is a highly complex phenomenon, and the vast variety of disease combinations makes it a difficult phenomenon to analyze. It is hardly practical to describe the prevalence and health outcomes of every conceivable disease combination, and much information is lost if multimorbidity is explored solely by counting disorders or applying one of several disease severity indices, for instance the Charlson Comorbidity Index. A less reductionist strategy involving a partitioning of the population into a limited number of subgroups with distinct disease pattern seems more promising and may provide a richer and more nuanced understanding of multimorbidity.

A growing body of epidemiological research focuses on patterns and clusters of chronic diseases including a number of population studies [11–20] and two recent reviews [21, 22]. Yet, compared with our knowledge of specific chronic diseases, our epidemiological knowledge of the prevalence and consequences of frequently occurring disease combinations remains limited. Further research is required to deepen our understanding of how multiple diseases tend to compound and interact. Studies of segments of the population with diverging disease profiles may give us more nuanced, segment-specific knowledge about prevention and treatment needs, social health disparities, and adverse impacts on quality of life and mortality. In addition, identifying common clusters of chronic diseases may enable policymakers and clinicians to simplify the care process for multimorbid patients and to better understand the reasons for poorer health in certain patient groups.

Given the many possible disease combinations, it is necessary to use advanced statistical techniques to segment the population into subgroups with similar disease profiles. A number of model and non-model-based clustering methods are available for this purpose [23]. The latter group includes traditional cluster analysis techniques (hierarchical cluster analysis, *k*-means clustering, etc.) which have been criticized for being descriptive, a-theoretical, and non-inferential [24]. For this study, we have chosen to use Latent Class Analysis (LCA). LCA is a model-based approach that seeks to identify homogeneous groups within a heterogeneous population by hypothesizing an unobserved categorical variable with n categories where each category represents a latent class [25]. Individuals in the same class share a common joint probability distribution among the observed variables (e.g. the same disease probability profile). Mathematically, LCA is closely related to factor analysis (FA), but LCA is considered preferable to FA for segmentation purposes [26]. FA rests on the assumption that a small number of latent variables (factors) are responsible for the covariances of the observed variables [26]. Hence, FA can be used for identifying disease clusters, but since these clusters are not related to groups, population segments need to be constructed post hoc on the basis of the estimated individual factor scores adding some extra steps to the analytical decisions the researcher must take.

To our knowledge, five recent studies on multimorbidity have applied LCA [11, 14–17]. None of them, however, are national studies covering all age groups from 16 and above. The first objective of the present study is to identify clusters of multimorbidity in the general population using LCA in a large, national, representative population study. The second objective is to examine how these clusters are associated with socio-demographic factors and health-related quality of life.

## Materials and Methods

### Setting and participants

Analyses in this study are based on data from the Danish national health survey coined “How are you?”, conducted in 2013 by the five Danish regions and the National Institute of Public Health at the University of Southern Denmark. “How are you?” is a national, representative, cross-sectional survey of the Danish population aged 16 years and over. It is based on a random sample of individuals with residence in Denmark as per 1 January 2013. The sample was drawn from the Danish Civil Registration System. A total of 300,450 individuals were invited to participate.

A mixed-mode approach was used to collect the data where each participant could fill out an enclosed questionnaire or use a unique web-access. Data were collected during the spring of 2013 using a maximum of three reminders. In all, 162,283 individuals participated in the survey giving a total response rate of 54%. Detailed information on the design and contents of a similar survey conducted in 2010 is reported elsewhere [27]. The questionnaire including all relevant questions is available online in Danish [28].

Prior to data analyses, respondents and non-respondents were linked to Danish national registers using the unique personal identification number given to all Danish citizens as a key. A weight was estimated to account for differences in selection probabilities and for differences in response rates for different sub-groups using a model-based calibration approach [29]. The weight was based on register information on sex, age, municipality of residence, educational level, income, marital status, country of birth, visits to the general practitioner, hospitalization, occupational status, and owner/tenant status for both responders and non-responders. This weight variable was added to the data set making it possible to weight data to represent the Danish population.

### Measures of chronic diseases

Data on 18 chronic conditions were collected using a revised version of a survey instrument recommended by the World Health Organization for national health surveys [30]. The conditions were selected because of their serious nature (potentially fatal and/or limiting daily activities) and high economic cost. Respondents were recorded as having a particular disease if they currently had the disease or if they had previously had the disease and still suffered from after-effects. Non-completed questions were considered as being disconfirmed if at least one of the questions on the chronic disease list was completed. The case was excluded from the analysis if none of the items on the list were completed. For the present analyses, some of the disease categories were combined to enhance the quality of data, producing a total of 15 disease categories. Multimorbidity was defined as having two or more of these 15 chronic diseases (see Table 1).

**Table 1:** Characteristics of the Study Population.

Characteristics	n	Weighted prevalence (%)
Gender		
Male	74,550	49
Female	87,733	51
Age (mean (SD))		47.76 (18.99)
Age (years)		
16–24	17,006	14
25–34	14,617	14
35–44	22,698	17
45–54	30,386	18
55–64	31,302	15
65–74	29,721	13
75+	16,553	9
Educational level		
Low	24,544	18
Medium	73,061	50
High	46,238	32
Cohabitation status		
Married/cohabitating	111,345	60
Single	50,938	40
Ethnic origin		
Danish	152,356	89
Other	9,927	11
Work status		
Working	88,907	58
Non-working	67,615	42
Diseases		
Hypertension	34,172	18
Ischemic heart disease	4,394	3
Stroke	2,819	2
Diabetes	9,202	5
Cancer	5,070	3
COPD	7,510	4
Asthma	11,098	7
Allergy	32,063	21
Arthritis	39,285	21
Osteoporosis	6,084	3
Slipped discs/other back injuries	21,660	13
Mental disorders	13,592	10
Migraine/recurrent headache	21,431	14
Tinnitus	20,295	12
Cataract	7,645	4
Multimorbidity (2+ chronic conditions)	64,349	37
Number of chronic conditions reported (mean (SD))		1.39 (1.49)

SD = standard deviation; COPD = chronic obstructive pulmonary disease

### Socio-demographic factors and health-related quality of life

Socio-demographic factors included gender, age, educational level, cohabitation, ethnic origin, and work status. Information on gender, age, and ethnic origin was collected from national registers to avoid missing data. All other data were self-reported. Educational level was categorized as either low (0–10 years), medium (11–14 years), or high (>15 years) based on information about completed primary, secondary, and higher education. Cohabitation status was categorized as married/cohabitating or single. Respondents were classified as Danish if, regardless of place of birth, they had at least one parent who was a Danish citizen born in Denmark. Work status was categorized as either working or non-working.

To measure functional status and health-related quality of life, the SF-12 instrument comprising 12 questions was used [31]. The SF-12 generates eight subscales that each measures a different dimension of health: The *Physical Functioning* scale describes whether health limits the ability to perform physical activities. The *Role Physical* scale covers limitations of physical health related to the kind and quality of work performed or other daily activities. The *Bodily Pain* scale describes the extent to which normal work activities are hampered by pain. The *General Health* scale describes the person’s self-rated health. The *Vitality* scale captures ratings of energy level. The *Social Functioning* scale measures the impact of either physical or emotional problems on social activities. The *Role Emotional* scale covers mental health-related role limitations. The *Mental Health* scale measures psychological distress and well-being. The eight subscales are calibrated to have an average of 50 and a standard deviation of 10 in the general US population (norm-based scoring), making it possible to meaningfully compare scores across domains.

### Ethics

The study was approved by the Danish Data Protection Agency (j. no: 2007-58-0010) and was undertaken in accordance with the Helsinki Declaration. The participants’ voluntary completion and return of the survey questionnaires constituted implied consent.

### Data analysis

The data analysis in the present study evolved over three steps: (1) identifying latent classes with different disease patterns in the general population; (2) analyzing associations between socio-demographic factors and latent class membership; and (3) analyzing variations in health-related quality of life across latent classes. All analyses were conducted using Latent GOLD 5.0 statistical software [32].

In the first step, LCA was employed to empirically identify patterns of multimorbidity by assigning individuals to a set of discrete, mutually exclusive groups—latent classes—based on their responses to the 15 chronic disease indicators. The assignment of an individual to a class is probabilistic rather than deterministic. We used LCA as an explorative technique (unconstrained LCA) with no a priori assumptions about the number of latent classes. Instead, a sequence of LCA models was estimated starting with a one-class model and increasing the number of classes in a stepwise fashion. In total, 15 models were fitted to the data. In order to ensure that global rather than local maxima were reached, we used an iterative maximum likelihood estimate with at least 500 random sets of starting values combined with an inspection of the corresponding log likelihood values. If necessary, the number of random sets was increased until the log likelihood had been replicated a minimum of five times.

Given that there is no single indicator reflecting an optimal model fit, model selection was based on a balance of parsimony, substantive consideration, and several fit indices. When determining the optimum number of classes in an LCA model [33], the following criteria are commonly used: (1) that there is an acceptable fit of the model to the data, (2) that the model is able to classify individuals into latent classes with a sufficient degree of accuracy, and (3) that the latent classes can be meaningfully interpreted, that is, it should be possible to assign a conceptually meaningful label to each class that distinguishes it from the other classes.

To measure the absolute fit of the estimated LCA models, chi-square goodness-of-fit tests are normally used. However, significance testing is problematic in the case of a large, sparse contingency table as well as a large sample size [34]. With sparse frequency tables, the asymptotic p-values associated with the chi-squared distribution are not valid. Chi-square goodness-of-fit tests also tend to reject a model when the sample size is large, even though the model is reasonable. In the present study with a sample size of over 150,000 and with 15 dichotomous indicators yielding 32,768 possible response patterns of which only 4,302 occurred in the sample, we encountered both a sparse table and a large sample. Instead of chi-square goodness-of-fit test, we therefore used the Index of Dissimilarity (I_d_) [35] and the Normed Fit Index (NFI) [36], which are both suitable for assessing model fit with sparse tables and/or large sample sizes. I_d_ takes the sample size into account, and values of I_d_ below 0.05 are generally considered to indicate a good fit. NFI is calculated by comparing the likelihood ratio chi-square of the model being tested with that of a baseline model. When a model accounts for 80–90% of the residuum variation, it is considered to have a good fit.

To measure the relative fit of the models, which refers to the adequacy of one model’s representation of data compared with that of another model, we used the Akaike Information Criterion (AIC) [37] and the Bayesian Information Criterion (BIC) [38]. Lower values on the AIC and the BIC indicate a better-fitting model. The BIC tends to select simpler models than the AIC, and in a Monte Carlo simulation it has been shown to be the most reliable criteria when deciding on the optimal latent class model [39]. Nonetheless, recent research has shown that even the BIC may result in more classes than are substantively useful [40].

The various measures of absolute and relative fit of the models were compared, and the substantive interpretation of each model was assessed before a final model was chosen.

As with any analysis, replication of the results of the present study would strengthen the findings. Therefore, the LCA was repeated in an independent sample from a previous Danish national health survey conducted in 2010 to test whether the same number of classes and similar disease profiles emerged.

In the second step of the analysis, we analyzed the association between socio-demographic characteristics and latent class membership. We conducted a bivariate analysis to describe the socio-demographic composition of the latent classes and a multivariate analysis to investigate how each variable predicted class membership using a multinomial logistic regression model with gender, age, educational level, cohabitation status, ethnic origin, and work status as covariates. The associations were evaluated using odds ratios (ORs) with 95% confidence intervals (CIs). Each odds ratio is adjusted for the remaining variables in the model.

In the third and final step, we examined the variation in health-related quality of life across latent classes by means of one-way analysis of variance (ANOVA) using scores from the eight SF-12 scales. For the second and third steps of the analysis, we used two newly developed, so-called three-step estimators implemented in LatentGOLD 5.0 to adjust for possible biases due to classification errors occurring when assigning subjects to their most likely class [41, 42].

## Results

### Sample description

The mean age of the sample population was 47.8 ± 19.0 years (range: 16–104 years) and the male proportion was 0.49. Of the 15 conditions included in the LCA, three had a prevalence >15%, four had a prevalence of 10% to 15%, while the remaining eight diseases had a prevalence <10%. See Table 1 for additional sample characteristics.

### LCA results

**Fig 1:**
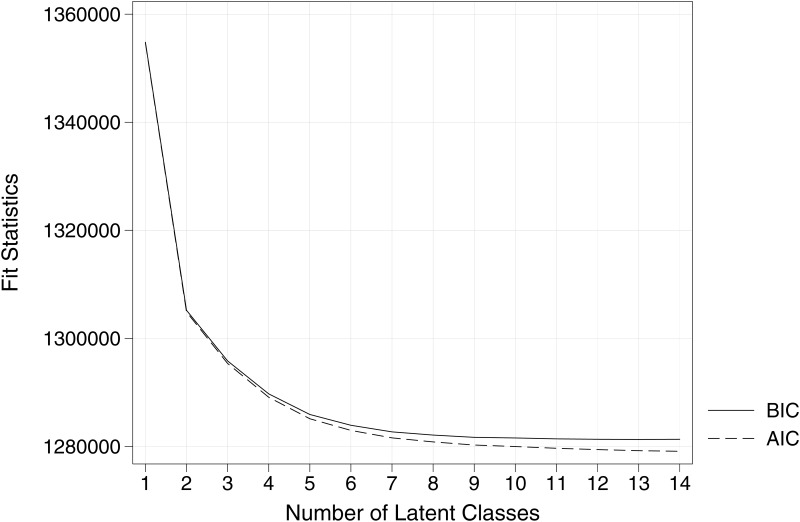
Relative Fit for Latent Class Analysis (BIC, AIC). BIC = Bayesian Information Criterion; AIC = Akaike Information Criterion

The LCA model fit results are summarized in Table 2. As for the relative goodness-of-fit indices, the value of AIC continued to decrease for the estimated models from the one-class to the fifteen-class model, whereas BIC reached a minimum in the thirteen-class model. However, there was no substantial improvement in either AIC or BIC fit beyond models with seven to eight classes; cf. the elbow-shaped curve in Fig 1. The I_d_ and the NFI further qualified the selection of a model. As classes were added to the one-class model, I_d_ decreased and NFI increased. In the seven-class model, I_d_ reached 0.05 and NFI reached 83%, suggesting an acceptable fit. Moreover, upon examination, the seven-class model appeared to have a meaningful interpretation. Consequently, based on a balance of several fit indices, parsimony, and model interpretability, the seven-class model was chosen as the final model. When cases are classified into clusters using the modal assignment rule, a certain amount of misclassification error is present. The total proportion of classification error is 23% for the seven-class model, which is considered acceptable.

**Table 2:** Fit Statistics for Latent Class Analyses.

Number of latent classes	Number of parameters estimated	LL	BIC	AIC	Classification error	Dissimilarity index
1	15	-677,317	1,354,813	1,354,664	0.00	0.237
2	31	-652,414	1,305,200	1,304,891	0.10	0.127
3	47	-647,631	1,295,825	1,295,356	0.17	0.095
4	63	-644,488	1,289,730	1,289,102	0.14	0.083
5	79	-642,485	1,285,917	1,285,129	0.21	0.065
6	95	-641,388	1,283,914	1,282,966	0.18	0.054
7	111	-640,681	1,282,692	1,281,584	0.23	0.050
8	127	-640,292	1,282,105	1,280,838	0.23	0.046
9	143	-639,985	1,281,683	1,280,256	0.26	0.043
10	159	-639,831	1,281,566	1,279,979	0.27	0.042
11	175	-639,653	1,281,402	1,279,656	0.31	0.040
12	191	-639,517	1,281,321	1,279,415	0.32	0.037
13	207	-639,402	**1,281,283**	1,279,218	0.31	0.035
14	223	-639,327	1,281,325	1,279,099	0.32	0.036
15	Not well identified					

LL = Log likelihood; BIC = Bayesian Information Criterion; AIC = Akaike Information Criterion

### Substantive interpretation

Class proportions and the estimated probabilities of having any particular chronic disease given membership of a latent class are shown in Table 3. In addition, the prevalence of multimorbidity and the average number of chronic conditions per individual are indicated within each class. It is a general feature of the seven-class model that it divides the population into one class without multimorbidity and six classes with a high prevalence of multimorbidity (81–100%), characterized by diverging disease profiles.

**Table 3:** Class Proportions and Class-Specific Probabilities from Seven-Latent-Class Model of Chronic Conditions.

	Latent Class						
Class	1	2	3	4	5	6	7
Assigned label	Relatively Healthy	Hyper-tension	Musculo-skeletal Disorders	Headache-Mental Disorders	Asthma-Allergy	Complex Cardio-metabolic Disorders	Complex Respira-tory Disorders
Class proportion	0.59	0.14	0.10	0.07	0.06	0.03	0.02
Item-response probabilities							
Hypertension	0.05	**0.63**	0.25	0.13	0.05	***0*.*73***	0.38
Ischemic heart disease	0.00	0.08	0.02	0.03	0.00	*0*.*30*	0.09
Stroke	0.00	0.05	0.01	0.02	0.00	*0*.*14*	0.04
Diabetes	0.01	0.23	0.02	0.02	0.01	*0*.*29*	0.12
Cancer	0.01	0.06	0.06	0.02	0.01	*0*.*09*	0.07
COPD	0.01	0.06	0.06	0.04	0.01	0.22	***0*.*69***
Asthma	0.02	0.02	0.01	0.08	0.46	0.16	***0*.*91***
Allergy	0.14	0.10	0.19	0.33	***0*.*94***	0.34	0.46
Arthritis	0.05	0.33	**0.77**	0.30	0.08	***0*.*84***	**0.50**
Osteoporosis	0.01	0.06	0.13	0.02	0.00	*0*.*19*	0.15
Slipped discs/other back injuries	0.05	0.09	0.37	0.35	0.08	***0*.*60***	0.30
Mental disorders	0.06	0.06	0.08	*0*.*42*	0.13	0.30	0.19
Migraine/recurrent headache	0.09	0.05	0.12	***0*.*65***	0.18	0.37	0.21
Tinnitus	0.07	0.16	0.21	0.22	0.09	*0*.*34*	0.20
Cataract	0.01	0.12	0.11	0.01	0.00	*0*.*26*	0.12
Multimorbidity (2+ chronic conditions) (%)	0.00	0.84	1.00	1.00	0.81	1.00	1.00
Number of chronic conditions reported (mean)	0.43	1.91	2.25	2.54	2.25	4.48	5.37

Item-response probabilities > 0.5 in **bold** to facilitate interpretation

Within each item, the class with the highest response probability is in *italic*

COPD = chronic obstructive pulmonary disease

*Class 1* was characterized by individuals with low probabilities of all 15 medical conditions when compared with all other classes. This group was labeled *Relatively Healthy*. The only condition with a probability of some size was allergy (14%). The prevalence of multimorbidity in Class 1 was 0%, and the average number of chronic conditions was 0.43. The majority of the sample (59%) was classified into the relatively healthy class.

*Class 2* was characterized by individuals who had a high probability of hypertension. Moreover, membership of the class was associated with an increased likelihood of diabetes and arthritis. Class 2 was labeled *Hypertension*. The prevalence of multimorbidity in Class 2 was 84%, and the average number of chronic conditions was 1.91. Fourteen percent of the sample was classified into this class.

*Class 3* was characterized by individuals who had a very high probability of arthritis. Indeed, members of Class 3 had a higher probability of arthritis than all other classes, except Class 6. Moreover, membership of Class 3 was associated with an increased probability of slipped discs/other back injuries, hypertension and osteoporosis. This class was labeled *Musculoskeletal Disorders*. The prevalence of multimorbidity in Class 3 was 100%, and the average number of chronic conditions was 2.25. Ten percent of the sample was classified into this class.

*Class 4* was characterized by individuals who had migraine/recurrent headache. Moreover, individuals in Class 4 also had higher probabilities of mental disorders than all other classes. Hence, the class was labeled *Headache-Mental Disorders*. Class 4 also had increased probabilities of allergy, arthritis, and slipped discs/other back disorders. The prevalence of multimorbidity in Class 4 was 100%, and the average number of chronic conditions was 2.54. Seven percent of the sample was classified into Class 4.

*Class 5* was characterized by individuals mainly affected by asthma and allergy and was labeled *Asthma-Allergy*. The probabilities of all other diseases were on the same level or slightly higher than in Class 1. The prevalence of multimorbidity in Class 5 was 81%, and the average number of chronic conditions was 2.25. Six percent of the sample was classified into Class 5.

*Class 6* was characterized by individuals with severe multimorbidity. Overall, there was a significantly higher morbidity in Class 6 than in Class 1 to Class 5. Ten out of 15 medical conditions had a higher probability of occurrence in Class 6 than in all other classes. One particularly distinctive feature of Class 6 was that membership of the class was associated with an increased likelihood of cardiometabolic disorders (diabetes, heart disease, stroke, and hypertension). Class 6 was therefore labeled *Complex Cardiometabolic Disorders*. The prevalence of multimorbidity in Class 6 was 100%, and the average number of chronic conditions was 4.48. Three percent of the sample was classified into Class 6.

Like Class 6, *Class 7* was characterized by individuals with severe multimorbidity, but members of this Class had respiratory diseases (asthma and chronic obstructive pulmonary disease (COPD)) as the most likely diseases. There was generally an increased probability of disease in this group. This class was labeled *Complex Respiratory Disorders*. The prevalence of multimorbidity in Class 7 was 100%, and the average number of chronic conditions was 5.37. Class 7 was the smallest of all classes, comprising 2% of the sample.

The analysis was replicated in an independent sample from the Danish health survey 2010 (N = 177,639) using the method described above. The LCA yielded substantially the same results as those presented in Table 3, that is, a seven-class model was the optimal choice, producing similar class prevalences and response probabilities.

### Posterior analysis

Table 4 shows the socio-demographic composition of the seven latent classes and the results of the multinomial logistic regression analysis. Class 1 (Relatively Healthy) and class 5 (Asthma-Allergy) were quite similar in terms of socio-demographic composition, as were also Class 6 (Complex Cardiometabolic) and class 7 (Complex Respiratory). However, considerable differences were seen between Class 1 and 5 on the one hand and Class 6 and 7 on the other hand. Individuals belonging to Class 1 and 5 were generally younger and better educated, and more were married or cohabiting and in employment than in Class 6 and 7 where the majority were older, non-working, and one-half was single. Class 2 (Hypertension) resembled Class 6 and 7 with respect to age distribution. Class 2 differed, however, from these two classes by having a higher proportion of men, a higher level of education, and higher labor force participation and marriage-cohabitation rates. Class 3 (Musculoskeletal) and Class 4 (Headache-Mental) were both characterized by having a high percentage of women, whereas Class 4 had a younger age composition than Class 3, with most being middle-aged.

**Table 4:** Demographics and Multinomial Logistic Regression Results for Covariates by Latent Disease Class.

	Latent class														
	Class 1 Relatively Healthy (Ref)		Class 2 Hypertension		Class 3 Musculoskeletal Disorders		Class 4 Headache-Mental Disorders		Class 5 Asthma-Allergy		Class 6 Complex Cardiometabolic Disorders		Class 7 Complex Respiratory Disorders		
Characteristics	%	OR	%	OR	%	OR	%	OR	%	OR	%	OR	%	OR	Wald test P-value
Gender															
Male	54	1.0	59	1.0	35	1.0	21	1.0	48	1.0	44	1.0	41	1.0	p<0.001
Female	46	1.0	41	0.7*	65	1.9*	79	2.8*	52	1.3*	56	1.3*	59	1.3*	
Age (mean)	38		68		64		45		35		68		65		p<0.001
Age (years)															
16–24	22		0		0		6		28		0		0		p<0.001
25–34	22		0		0		14		23		0		0		
35–44	23		0		2		32		23		2		3		
45–54	18		11		18		36		15		10		15		
55–64	10		24		31		13		7		26		28		
65–74	4		35		29		0		3		29		28		
75+	1		31		19		0		1		34		25		
Age (per 5-year increase)		1.0		2.4*		2.1*		1.3*		0.9*		2.0*		1.9*	
Educational level															
Low	12	1.0	28	4.4*	21	2.8*	21	2.6*	14	1.0	42	9.6*	37	5.7*	p<0.001
Medium	50	1.0	50	2.0*	50	1.6*	51	1.5*	50	1.0	43	2.9*	44	1.9*	
High	37	1.0	22	1.0	30	1.0	28	1.0	36	1.0	14	1.0	19	1.0	
Cohabitation status															
Married/cohabitating	62	1.0	66	1.0	71	1.0	63	1.0	58	1.0	46	1.0	53	1.0	p<0.001
Single	38	1.0	34	1.2*	29	1.1*	37	1.0	42	1.0	54	2.2*	47	1.9*	
Ethnic origin															
Danish	88	1.0	94	1.0	96	1.0	80	1.0	90	1.0	85	1.0	89	1.0	p<0.001
Other	12	1.0	6	1.1	4	0.8*	20	1.4*	10	0.8*	15	2.7*	11	1.4*	
Work status															
Working	73	1.0	22	1.0	31	1.0	51	1.0	71	1.0	9	1.0	14	1.0	p<0.001
Non-working	23	1.0	74	2.2*	64	2.1*	45	3.4*	25	1.1	88	20.4*	83	6.4*	

* = *p*<0.05

OR = odds ratio. Each odds ratio is adjusted for the remaining variables in the model.

The multivariate, multinomial logistic regression model showed that being female increased the likelihood of belonging to any of the six multimorbidity classes, except for Class 2 (Hypertension). In particular, women had an increased likelihood of belonging to Class 4 (Headache-Mental). Higher age strongly increased the likelihood of belonging to Class 2 or 3 and Class 6 or 7 (Hypertension, Musculoskeletal, Complex Cardiometabolic, and Complex Respiratory). Low educational attainment predicted membership of any of the multimorbidity classes, except Class 5 (Asthma-Allergy). In particular, it was found that low education strongly increased the likelihood of belonging to Class 6 or 7. Furthermore, being single increased the likelihood of membership of Class 6 or 7. Ethnic origin only moderately predicted class membership. Individuals with another ethnic origin than Danish had a somewhat lower likelihood of belonging to Class 3 (Musculoskeletal) or 5 (Asthma-Allergy), and a somewhat higher likelihood of belonging to Class 4 (Headache-Mental) and Class 6 or 7 (Complex Cardiometabolic and Complex Respiratory). Having a non-working status was a predictor of membership of any of the six multimorbidity classes except Class 5 (Asthma-Allergy). An especially strong association was seen between non-working status and membership of Class 6 or 7 (note that a majority in these two classes had passed the age of retirement).

**Fig 2:**
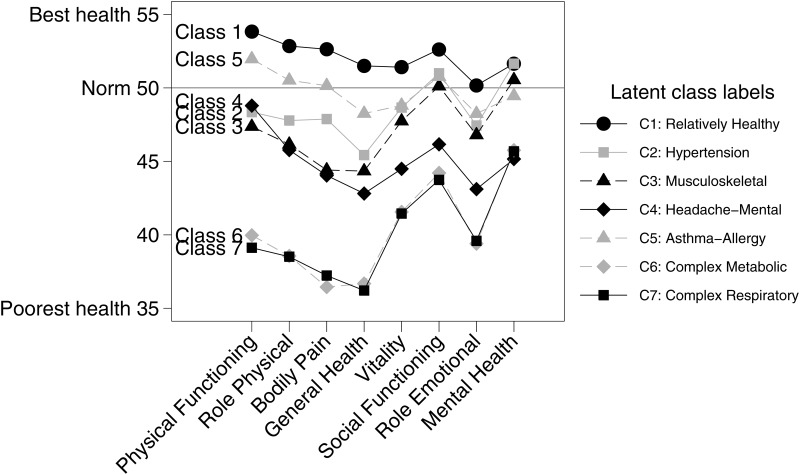
Self-Reported Health Status Stratified by Class.

The variation in health-related quality of life across latent classes is reported in Fig 2.

Mean values for the eight SF-12 scales are presented as a profile for each of the latent classes, with the four scales most closely associated with physical health on the left side of the x-axis and the four scales most closely associated with mental health on the right side. Generally, there were marked differences in health-related quality of life between individuals belonging to the seven latent classes. As could be expected, Class 1 (Relatively Healthy) was the class with the best health status. The greatest contrast was found between Class 1 and the two classes characterized by severe multimorbidity, Class 6 (Complex Cardiometabolic) and Class 7 (Complex Respiratory). Despite different disease patterns, Class 6 and Class 7 had nearly identical profiles, with scores substantially below the norm on the physical health scales and, though to a lesser extent, on the mental health scales. Class 5 (Asthma-Allergy) had a profile that was fairly similar in shape to that of Class 1, but with a slightly lower score on all the scales. Class 2 (Hypertension) and Class 3 (Musculoskeletal) had average scores well below the norm on the four physical health scales (Class 3 more so than Class 2), whereas their mean scores on the mental health scales were somewhat higher with profiles resembling those of Class 5. Class 4 (Headache-Mental) had a profile on the four physical scales similar to that of Class 3, but it had substantially lower scores on the four mental health scales.

## Discussion

The present study examined chronic disease patterns in the general Danish population using LCA. Seven latent classes were identified, one class without multimorbidity, which included 59% of the population, and six classes with a high prevalence of multimorbidity. Use of the LCA model substantially reduced data complexity since more than 4,000 observed disease combinations were reduced to a limited number of latent classes. Moreover, this approach allowed us to uncover important differences between subgroups of the multimorbid population. The population segments belonging to the six multimorbidity classes had different disease profiles, and the disease burden varied considerably between the classes, both qualitatively (type of diseases) and quantitatively (number and prevalence of diseases). Overall, individuals with multimorbidity were older, less educated, and had a poorer health-related quality of life; and multimorbidity was more prevalent among women than among men. However, we found significant differences in the socio-demographic composition and the health-related quality of life between the six multimorbid classes.

Class 6 (Complex Cardiometabolic) and Class 7 (Complex Respiratory), which together comprised 5% of the population, carried the heaviest disease burden. Although they differed from one another in terms of disease profiles, they had a significantly higher average number of diseases than the remaining multimorbidity classes. Both groups had a mixture of potentially fatal and non-fatal, but quality-of-life-impairing diseases. Compared with Class 2 (Hypertension) and Class 3 (Musculoskeletal), which had similar age profiles, but a much smaller burden of disease, the educational level was lower in Class 6 and 7, which indicates a social gradient in severe multimorbidity. Health-related quality of life was poor in Class 6 and 7 in general and specifically so compared with the similar-aged Class 2 and 3. Overall, Class 6 and 7 form a population segment with complex health and social care needs requiring comprehensive coordination and patient/caregiver involvement to counter fragmentation of services and minimize the burden of treatment and side effects.

Noteworthy is also the imbalanced gender composition within the seven latent classes with a predominance of men in Class 1 (Relatively Healthy) and Class 2 (Hypertension) and a predominance of women in the remaining classes. The female predominance is outspoken in Class 3 (Musculoskeletal), and even more so in Class 4 (Headache-Mental), which points to gender-specific differences in life course trajectories of health. Our findings confirm that women are more prone than men to suffer from musculoskeletal disorders, depression, and headache as stated in several epidemiological surveys [43–45]; likewise, our study confirms the significance of gender differences in multimobidity patterns [20]. Moreover, our findings add to existing knowledge about common chronic diseases by showing that particular ailments often coexist, for instance headache and mental disorders, and that they occur with other diseases, too. This knowledge may inform the design of holistic health-promoting activities aiming to prevent sickness absence and labor market exclusion. It may also inform medical and vocational rehabilitation initiatives, and our finding warrant that particular focus be devoted to preventing women’s premature exit from the labor force.

Age is generally a strong correlate of multimorbidity. Another notable finding of the present study is therefore that a single class, Class 5 (Asthma-Allergy), has an age profile dominated by individuals under 45 years. Although several studies have shown that multimorbidity is not only a problem of old age [46–48], multimorbidity among young adults remains an under-researched area. Even though individuals belonging to Class 5 generally have a much better functional health status than the other multimorbidity classes, recent research suggests that asthma, which is highly prevalent in this class, and COPD may share similar pathogenic mechanisms [49]. This may predispose individuals with asthma for COPD later in life with smoking as the major mediating risk factor [50]; this finding underlines the need for smoking prevention and cessation interventions targeting this population segment.

### Previous studies on multimorbidity patterns

The findings in the present study are not easily compared with previous LCA findings of multimorbidity because earlier studies cover more limited population segments and/or different disease spectra. Prados-Torres and colleagues identified 14 articles on general patterns of *associative multimorbidity* (i.e. non-random association between diseases) in a recent systematic review [21]. Although the studies reviewed exhibited considerable methodological heterogeneity and used different statistical procedures (cluster analysis, factor analysis, etc., but not LCA), the authors found three general patterns dominated by cardiovascular and metabolic diseases, mental health problems, and musculoskeletal disorders, respectively. These patterns recurred in all studies among the otherwise large number of disease patterns studied. These apparently robust findings are consistent with Class 6, 3 and 4 in the present study. Our study adds to this review by demonstrating, firstly, a segment dominated by hypertension (Class 2), which could be hypothesized to consist of people at high risk of developing complex cardio-metabolic disorder later in life (Class 6), and, secondly, two segments with allergy and respiratory disorders (Class 5 and 7).

### Limitations

To the best of our knowledge, the present study is the first to examine latent classes of a large number of chronic diseases in a large, national, representative sample across a broad age span. Still, a number of limitations should be mentioned. First, the cross-sectional nature of the data used implies that no conclusions about temporality or causation between the chronic diseases investigated can be made; longitudinal analysis over an extended period is needed to estimate the incidence of transitions between latent classes and to identify characteristics associated with the development of multimorbidity of increasing severity [51]. Second, the study was based on a set of self-reported diseases. Hence, the patterns of multimorbidity may have been different if clinical data or other chronic diseases had been included. However, using self-reported data allowed us to obtain information about diseases that are commonly excluded in studies that rely on register data (e.g., allergy, migraine, and musculoskeletal diseases). Third, our study included 15 chronic diseases selected because of their serious nature (potentially fatal and/or limiting daily activities) and high economic cost. However, respondents may have suffered from other non-listed chronic diseases. To heighten the external validity, it is recommended that future studies include more chronic diseases. Finally, the response rate among the oldest old was rather low, and people who were very burdened by chronic diseases may not be adequately represented. Also, people who had limited Danish language skills may not have participated in the survey. This may have introduced selection and information bias. Yet, the population weights compensate for non-response and differences in selection probabilities.

Despite these limitations, the findings of the present study clearly support the relevance of investigating patterns of multimorbidity using LCA. Furthermore, replication of the results in an independent sample strengthened confidence in the generalizability of the findings.

### Conclusion and implications

The present study demonstrates that the general population consists of segments characterized by distinct disease patterns. The insight we have gained by opening up the black box of multimorbidity can be used to design more efficient treatment and prevention strategies. At the clinical level, this knowledge calls for a differentiated treatment strategy for patients belonging to each of the six multimorbidity classes. At the population level prevention and public health strategies should similarly be informed by knowledge of the disease segments.

From a clinical perspective one of the main challenges associated with multimorbidity is to avoid an excessively high treatment burden. Meeting the healthcare needs of individuals with severe multimorbidity challenges the present healthcare system, which is characterized by a high degree of fragmentation and specialization. The importance of matching the demands imposed by treatment with the capacity of the patient is stressed by the fact that five out of six multimorbidity classes had substantial physical and mental functional deficits compared with the relatively healthy group—most so in the subgroups with complex metabolic and respiratory conditions. Moreover, the multimorbid segments generally had less favorable socio-demographic characteristics (higher age, lower educational level, more singles, and non-working persons) than the relatively healthy group. It seems therefore obvious that effective, appropriate, and good-quality care for multimorbid patients must move “beyond silos” towards integrated healthcare and social care.
